# Linkage disequilibrium and genome-wide association analysis for anthocyanin pigmentation and fruit color in eggplant

**DOI:** 10.1186/1471-2164-15-896

**Published:** 2014-10-14

**Authors:** Fabio Cericola, Ezio Portis, Sergio Lanteri, Laura Toppino, Lorenzo Barchi, Nazzareno Acciarri, Laura Pulcini, Tea Sala, Giuseppe Leonardo Rotino

**Affiliations:** Dipartimento di Scienze Agrarie, Forestali e Alimentari (DISAFA) - Plant Genetics and Breeding, University of Torino, Largo P. Braccini 2, I-10095 Grugliasco, Torino Italy; Consiglio per la Ricerca e Sperimentazione in Agricoltura - CRA-ORL, Research Unit for Vegetable Crops, I-26836 Montanaso Lombardo, Lodi, Italy; Consiglio per la Ricerca e Sperimentazione in Agricoltura - CRA-ORA, Research Unit for Vegetable Crops, I-63030 Monsampolo del Tronto, Ascoli Piceno, Italy

## Abstract

**Background:**

The genome-wide association (GWA) approach represents an alternative to biparental linkage mapping for determining the genetic basis of trait variation. Both approaches rely on recombination to re-arrange the genome, and seek to establish correlations between phenotype and genotype. The major advantages of GWA lie in being able to sample a much wider range of the phenotypic and genotypic variation present, in being able to exploit multiple rounds of historical recombination in many different lineages and to include multiple accessions of direct relevance to crop improvement.

**Results:**

A 191 accessions eggplant (*Solanum melongena* L.) association panel, comprising a mixture of breeding lines, old varieties and landrace selections originating from Asia and the Mediterranean Basin, was SNP genotyped and scored for anthocyanin pigmentation and fruit color at two locations over two years. The panel formed two major clusters, reflecting geographical provenance and fruit type. The global level of linkage disequilibrium was 3.4 cM. A mixed linear model appeared to be the most appropriate for GWA. A set of 56 SNP locus/phenotype associations was identified and the genomic regions harboring these loci were distributed over nine of the 12 eggplant chromosomes. The associations were compared with the location of known QTL for the same traits.

**Conclusion:**

The GWA mapping approach was effective in validating a number of established QTL and, thanks to the wide diversity captured by the panel, was able to detect a series of novel marker/trait associations.

**Electronic supplementary material:**

The online version of this article (doi:10.1186/1471-2164-15-896) contains supplementary material, which is available to authorized users.

## Background

Eggplant (*Solanum melongena* L.) ranks third in commercial importance among the solanaceous crops after potato and tomato, and it is cultivated in many countries, particularly in southern Asia, middle East and Northern Africa. Global production in 2012 was about 48 Mt [1], and the largest European producer is Italy. Despite its commercial importance, little research effort has been devoted to the genetic analysis of key breeding and quality traits with respect to the other Solanaceae crops tomato, potato and *Capiscum* [2–4]. Several quantitative trait loci (QTL) underlying fruit color and shape have been described by Nunome et al. [5], while Doganlar et al. [6] and Frary et al. [7] succeeded in identifying QTL for certain fruit- and plant-related traits, but only in a population derived from an inter-specific cross, which is of reduced utility in eggplant improvement. More recently, the genetic basis of parthenocarpy [8], *Ralstonia solanacearum* resistance [9], anthocyanin content [10] and a group of agronomic traits [11] has been elucidated.

The genome-wide association (GWA) approach represents an alternative to biparental linkage mapping for the determination of the genetic basis of traits [12]. Both approaches rely on recombination to re-arrange the genome [13], and seek to establish correlations between phenotype and genotype, based on the non-random association of alleles at two or more loci, termed *linkage disequilibrium* (LD). In a bi-parental population, only the polymorphisms between the two parents can be queried, whereas in a GWA population the number of polymorphisms is determined by the genetic diversity of the entire germplasm panel. In a GWA population, LD is determined not only by recombination frequency, but also by genetic drift, by the mating system of the plant and by the history of selection (reviewed by Rafalski and Morgante [14]). The major advantages of GWA over biparental linkage mapping lie in the much wider variability in phenotype and genotype made accessible, a history of multiple rounds of recombination in many different lineages and the inclusion of germplasm of direct relevance to crop improvement. The real risk that the genetic architecture of the germplasm panel may cloud the analysis has to be allayed by a prior evaluation of the population’s structure [15].

To date only one GWA-based study of variation in eggplant has been performed [16] but the present study used a larger number of accessions (191 *vs* 141*)* and a more densely populated genetic map (384 SNPs (single nucleotide polymorphisms) *vs* 105 microsatellites) and targeted traits related to anthocyanin accumulation and fruit color.

## Methods

### Plant material and DNA isolation

A core set of 191 accessions (Additional file 1: Table S1), chosen from a large collection of breeding lines, old varieties and landrace selections by Cericola et al. [17], was established to represent germplasm grown in east Asia (EA accessions) and in the Mediterranean basin (WE). The entries were all highly homozygous and thus phenotypically stable. Genomic DNA was extracted from fresh young leaves harvested from three individuals of each accession, using an E.Z.N.A.™ Plant DNA mini kit (OMEGA Bio-Tek, *Norcross*, *GA, USA*), according to the manufacturer’s protocol. The quality of each DNA sample was monitored by electrophoresis through an 0.8% agarose gel and its DNA concentration estimated spectrophotometrically (DU730, Beckman Coulter Brea CA, USA).

### SNP data acquisition

Each accession was genotyped at 384 SNP loci as reported by Barchi et al. [18]; 339 of these have been genetically mapped [10]. SNPs were selected taking into account a quality score, based on the probability of good performance using the Illumina Golden Gate assay (Illumina, San Diego, CA, USA); the score >0.6 indicates a high probability of success. A BlastX search was carried out against the TAIR9 dataset using the 2,201 highest quality score SNPs as query; the 384 sequences having the highest e-value were then chosen. The GoldenGate assay was carried out at the UC Davis Genome Center (http://www.genomecenter.ucdavis.edu). Automatic allele calling was handled by GenCall software (Illumina). Two of the entries were included in duplicate as an internal control. SNP loci in which the minimum allele frequency (MAF) fell below 5%, along with those where >10% of the data were missing, were discarded. Each SNP locus was scored as a binary data point, and the PIC (polymorphism information content) of each was estimated following Anderson et al. [19].

### Population structure

Genetic similarities between pairs of entries were quantified by the Dice [20] similarity index, then used to describe genetic relationships using both the unweighted pair-group arithmetic mean (UPGMA) method, and principal coordinate analysis (PCoA) by means of *Past 2.08* software [21]. *STRUCTURE v2.1* software [22] was used to estimate the number of sub-populations in the panel, applying the admixture model for the ancestry of individuals and correlated allele frequencies. The population structure was modelled with a burn-in of 50,000 cycles followed by 100,000 Markov Chain Monte Carlo (MCMC) repeats. The Evanno et al. [23] transformation method was then used to infer *K,* the most likely number of populations. Pair-wise kinship coefficients between the accessions were estimated using *SPAGeDi* software [24]. The diagonal of the matrix was set to two, and negative values were set to 0, following Yu et al. [15].

### LD analysis

LD decay was quantified by plotting pair-wise r^2^ values against the distance (cM) between adjacent SNP loci, based on the genetic map developed by Barchi et al. [10]. The effect of population structure on LD was investigated with three approaches as suggested by Mangin et al. [25]: r^2^ (an estimate of LD between SNP loci without any correction); r^2^_s_ (taking into account population structure derived from *STRUCTURE* analysis) and r^2^_sv_ (taking into account both the *STRUCTURE* output and the kinship matrix). To quantify the reach of LD, an r^2^ threshold of 0.15 was set [26]. The relationship between the baseline r^2^ values and genetic distance was determined using a locally weighted scatter plot smoothing line [27]. To visualize LD throughout the genome, heat maps were produced based on pair-wise r^2^, r^2^_s_ and r^2^_sv_ values [28]. The estimation of all LD measures was carried out by programs implemented in the *R* package *LDcorSV* [25].

### Acquisition and analysis of phenotypic and morphological data

The accessions were grown in field both at Montanaso Lombardo (ML: 45°20'N, 9°26'E) and at Monsampolo del Tronto (MT: 42°53'N; 13°47'E) in 2010 and again in 2011. In each trial, the material was set out as two randomized complete blocks with six plants per entry per block, and standard horticultural practices were applied. Phenotyping methodology was based on the European Cooperative Programme for Plant Genetic Resources Solanaceae descriptors (ECPGR [29]) and the International Board for Plant Genetic Resource descriptors for eggplant (IBPGR [30]). The traits assayed were adaxial leaf lamina anthocyanin (*adlan*), stem anthocyanin (*stean*), abaxial leaf lamina anthocyanin (*ablan*), calyx anthocyanin (*calan*), corolla color (*corcol*), adaxial leaf venation anthocyanin (*adlvean*), abaxial leaf venation anthocyanin (*ablvean*), peduncle anthocyanin (*pedan*), fruit color (*fcol*) and fruit glossiness (*fglo*). The anthocyanin content of the vegetative part of the plant was scored on a 0–5 scale, with “0” representing no visible anthocyanin pigmentation (completely green tissue), and “5” representing dark violet tissue. *corcol* was scored as “0” for white, “1” for pink and “2” for violet. *fcol* was measured using a CR-400 Chroma-meter (Konica Minolta, Tokyo, Japan) to generate three Hunter color coordinates (L*, a* and b*), averaged across three regions of the surface on each fruit; the measurements were reduced to a single variable by calculating the Euclidean distance from white (L* =100, a* =0, b* =0), following Prohens et al. [31]. *fglo* was scored as “1” for opaque, “2” for intermediate and “3” for bright peel color. The trait data were treated as adjusted entry means (best linear unbiased predictors). Several multivariate linear mixed models were tested using a combination of the F-test (for the fixed component) and the Akaike test (for the random component). The model shown to best fit the data was: *p*_*ijsb*_ *= l*_*j*_ *+ y*_*s*_ *+ r*_*bjs*_ *+ g*_*i*_ *+ m*_*ij*_ *+ n*_*is*_ *+ e*_*ijs*_, where *p*_*ijsb*_ represents the phenotype of the *b*^*th*^ replicate of the *i*^*th*^ genotype at the *j*^*th*^ location in the *s*^*th*^ year; *l*_*j*_ represents the fixed effect of the *j*^*th*^ location, *y*_*s*_ the fixed effect of the *s*^*th*^ year, *r*_*bjs*_ the fixed effect of the *b*^*th*^ replicate within the *j*^*th*^ location in the *s*^*th*^ year, *g*_*i*_ the random effect of the *i*^*th*^ genotype, *m*_*ij*_ the random effect of the genotype by location interaction, *n*_*is*_ the random effect of the genotype by year interaction and *e* the residual. Broad-sense heritabilities were calculated from the expression


where *σ*^2^_*g*_ represented the genotypic variance, *σ*^2^_*y*_ the genotype x year interaction, *σ*^2^_*l*_ the genotype x location interaction, *σ*^2^_*e*_ the residual variance, *n*_*y*_ the number of years (2) and *n*_*l*_ the number of locations (2). Pearson correlation coefficients were calculated between each pair of phenotypic traits. All analyses were carried out by algorithms implemented in *R* software [32].

### GWA mapping

The GWA analysis was performed using *Tassel v4.0.25* software [33]. Three models were tested: the simple general linear model (GLM, Naive-model), the structured association model (GLM, Q-model), based on the *STRUCTURE* output, and the mixed linear model (MLM, K + Q-model), taking into account both the *STRUCTURE* output and the kinship matrix [15]. The mixed-model approach has been used elsewhere [34–36] to analyse variation in qualitative traits by treating them as quantitative ones, on the assumption that averaging across replicates would produce normality. The cumulative density function was applied to assess the efficiency of the various models in correcting for population structure. The false positive rate (p-value) was converted into a false discovery rate [37], using the *QVALUE* package implemented in *R*. The estimation of the overall proportion of true null hypothesis π0 was based on λ range set from 0 to 0.95 by 0.05 and the smoother method was applied [38]. q-values <0.05 were considered as significant. For each significantly associated SNP locus, a general linear model with all fixed effect terms was applied to estimate the proportion of the phenotypic variance explained (PVE). In order to visualize the associations and to compare them with established QTL [6, 10], all SNPs associated with a particular trait mapping within less than double the mean LD stretch were considered as a single unit defining association groups. The resulting genetic map, incorporating the associations and QTL detected here into an F2-based linkage map [10], was drawn using *MapChart v2.1* software [39]. Synteny between tomato and eggplant chromosomal regions was investigated by aligning the RAD tag sequences [40] surrounding SNPs against the tomato SL2.40 genome sequence (http://solgenomics.net/) using the Burrows-Wheeler alignment tool [41]. Alignments with a MAPping Quality value >10 were considered as valid.

## Results

### Genotypic characterization and population structure

The 191 accessions were initially genotyped at 384 SNP loci, of which 338 were retained after quality control. The two replicated accessions gave uniformly consistent allele calls. MAF at most of the SNP loci ranged from 10% to 50% (Additional file 2: Figure S1A), with only 24 displaying a MAF value <5%. These were discarded, leaving a genotypic matrix of 191 entries by 314 SNP loci, of which 307 have been placed on the Barchi et al. [10] genetic map. The global average PIC value was 0.41; loci on chromosome E02 had a low mean PIC value (0.28), while the mean PIC for the remaining loci on a chromsome-by-chromosome basis lay between 0.38 and 0.46 (Additional file 2: Figure S1B).

**Figure 1:**
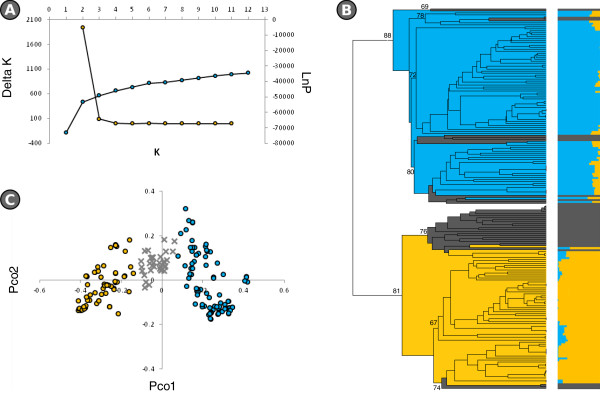
**The genetic architecture of the full germplasm panel. A)** Ln(K) and DK plots derived from the SNP data. **B)** UPGMA dendrogram derived after taking account of the STRUCTURE analysis. **C)** PCoA visualization of the genetic relationships between members of the association panel. Cluster A is shown in orange, cluster B in blue and admixtures in grey.

The *STRUCTURE* analysis resulted in a prediction for *K* of either 1 or 2 (Figure 1A). The UPGMA-based dendrogram (Figure 1B) and the PCoA (Figure 1C) show the genetic relationships between the 191 accessions. Their form, as well as the ΔK analysis provided by the Evanno et al. [23] transformation, suggested a population structure comprising two subgroups. According to the level of membership provided by *STRUCTURE*, cluster A contained 91% of the EA accessions, while 96% of the cluster B membership comprised WA accessions. The remaining 35 accessions (18%) had ambiguous membership and were thus classified as admixed. PCoAs carried out separately on the EA accessions showed some clustering among the Chinese entries, and among the Indian and SE Asian ones (Additional file 3: Figure S2A). The WA entries were grouped according to previously described morphology-based groups (Additional file 3: Figure S2B) [17], where group 1 accessions produced long, light and curved fruits, group 2 oblong shaped fruits of medium weight and group 3 round, heavy fruits.

### LD evaluation

**Figure 2:**
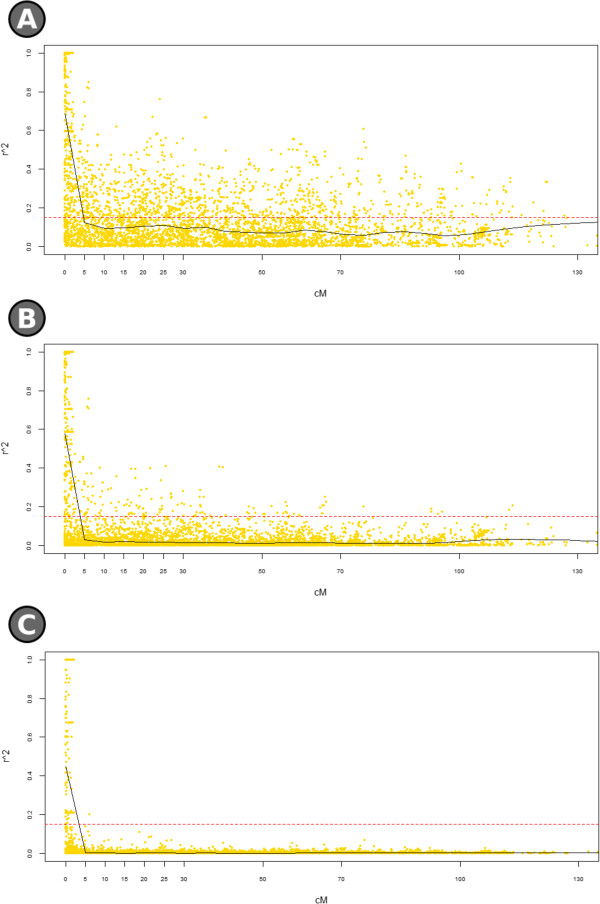
**LD decay.** The curve was fitted using a locally weighted scatterplot smooth regression with the threshold set at 0.15. **A)** r^2^ model, **B)** r^2^_s_ model, **C)** r^2^_sv_ model.

**Figure 3:**
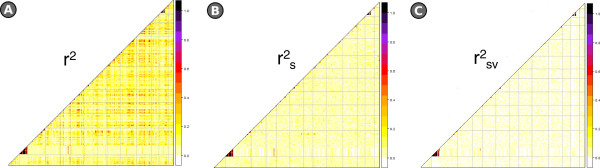
**Heat maps indicating genome-wide variation in LD across the genome. A)** r^2^ model, **B)** r^2^_s_ model, **C)** r^2^_sv_ model.

An r^2^ threshold of 0.15 was applied to define which SNP loci were significantly associated with one another. On the basis of the r^2^ model (with no correction for the population structure, Figure 2A), the mean genetic length of these associated groups was 4.8 cM. A mean r^2^ of 0.15 was observed between all pairs of linked loci, with a mean maximum r^2^ value of 0.56. The mean LD between unlinked loci was 0.10. When the r^2^_s_ model was applied (Figure 2B), the LD stretch was reduced to 3.9 cM, with a mean level of 0.07 between adjacent loci; an average of the highest r^2^_s_ value of each marker with any other of 0.45, and the mean LD between unlinked loci was 0.02. On the basis of the r^2^_sv_ model (Figure 2C), LD extended over 3.4 cM, with a whole genome mean of 0.03 between adjacent (maximum 0.26) and a mean LD between unlinked loci of less than 0.01. Heat maps produced for each of the three models showed that strong LD was limited to certain regions, mostly aligned to the diagonal (Figure 3). Apparent high levels of LD between loci mapping to a different chromosome were suggested in the r^2^ model, but this phenomenon was largely absent in the r^2^_s_ and r^2^_sv_ models.

### Phenotypic data analysis and association mapping

A summary of the accessions’ phenotypic performance is presented in Table 1 together with the heritabilities for scored traits. The PVEs are included in Additional file 4: Figure S3. A wide range of variation was observed for most of the traits, and the genotypic variance component was substantial for all of them (P <0.01). *pedan*, *fcol* and *calan* were the most genetically variable of the traits (Additional file 4: Figure S3), and also the most highly heritable. The GxE component of the variance (involving both season and location) was small, with the exception of that for *corcol* and *fglo*, the two least heritable of the traits. Broad-sense heritabilities averaged 0.71 (0.38-0.98), with the least heritable traits being *fglo* and *corcol*, and the most heritable *pedan* and *fcol* (Table 1). Some significant inter-trait correlations were observed: traits associated with the anthocyanin content of the plant (*adlan*, *stean*, *ablan*, *calan*, *adlevan*, *ablevan* and *pedan*) were strongly and positively correlated with one another, but only moderately correlated with *corcol*. The traits *fglo* and *fcol* were somewhat inter- correlated, and correlated with the anthocyanin content-related traits, but were uncorrelated with *corcol.* Both the correlation data and the distribution of each trait are reported in Additional file 5: Figure S4.

**Table 1:** **Codes used to identify the various traits along with mean values, standard deviations (SD), coefficients of variation (CV) and broad-sense heritabilities**

Trait	Code	Average	SD	CV	Heritability
Adaxial leaf lamina anthocyanin (scale 0–5)	*adlan*	1.37	1.49	1.09	0.67
Stem anthocyanin (scale 0–5)	*stean*	2.80	1.83	0.65	0.81
Abaxial leaf lamina anthocyanin (scale 0–5)	*ablan*	1.08	1.08	1.00	0.55
Calyx anthocyanin (scale 0–5)	*calan*	2.74	1.75	0.64	0.87
Corolla color (scale 0–3)	*corcol*	1.49	0.51	0.34	0.42
Adaxial leaf venation anthocyanin (scale 0–5)	*adlvean*	2.27	1.53	0.67	0.75
Abaxial leaf venation anthocyanin (scale 0–5)	*ablvean*	2.26	1.78	0.78	0.76
Fruit peduncle anthocyanin (scale 0–5)	*pedan*	2.20	2.13	0.97	0.95
Fruit color (L*a*b* coordinates distance from 0)	*fcol*	74.57	15.60	0.21	0.98
Fruit glossiness (scale 0–3)	*fglo*	2.37	0.73	0.31	0.38

**Figure 4:**
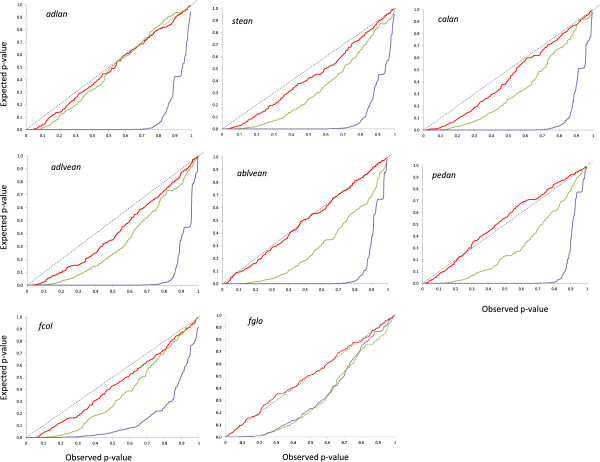
**Cumulative density function using three alternative association models: the GLM Naive (violet trace), GLM Q-model (green trace) and MLM (red trace).** Traits showing significant associations are indicated. The latter provided the most consistent p-values.

**Figure 5:**
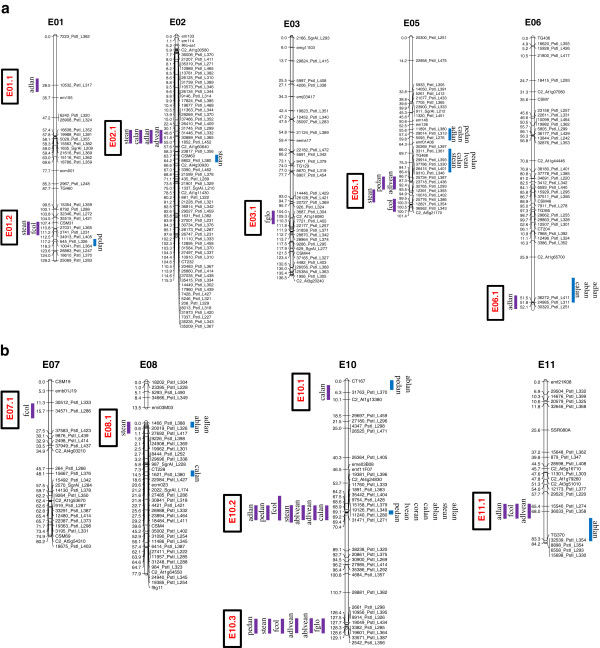
**Regions identified by GWA in comparison to QTL locations described by Barchi et al. **[10]**.** The GWA outcome is given to the left of each chromosome (the vertical bars represent a ±3.4 cM interval around the position of the associated SNP loci) and the various association groups are indicated in panels and marked in red. The QTL locations are shown to the right of each chromosome.

Associations between SNP alleles and morphology were acquired on the basis of three different models. The GLM Naive-model, which involves no correction for population structure, identified several spurious associations. This failing was improved by applying the GLM Q-model, but only the MLM K + Q-model produced a distribution of p-values comparable to the theoretical one (Figure 4). Thus the latter model was pursued. Following q-value correction, 56 significant genotype/phenotype associations were detected. Regions carrying the presumed genes/QTL were identified on nine of the 12 chromosomes (none were detected on chromosomes E04, E09 or E12) (Table 2), and involved eight of the ten traits (no associations involved either *ablan* or *corcol*). The number of associations per trait ranged from four (*calan* and *fglo*) to 11 (*stean*), and the total number of SNP loci involved was 20; these loci had a mean MAF of 33.4%. The PVE per association laid between 5% and 24% (mean 10%). In order to match the associations with previously identified QTL, loci linked to one another by <6.8 cM were considered as a unit, and their genomic location was obtained from the Barchi et al. [10] map. Overall, 12 association groups, comprising 1–4 SNP loci, were defined in this way (Figure 5). The most prominent clustering of traits occurred on chromosome E10, which also proved to harbor the most genes/QTL underlying variation in anthocyanin content and fruit color. The E10.2 group (four SNPs) harbored genetic factors for *adlan, stean, calan*, *adlevean, ablevean, pedan* and *fcol* while E10.3 (three SNPs) included genetic factors for *stean, ablevean*, *adlevean, pedan, fcol* and *fglo.* One of the two other large clusters was on chromosome E02 (one SNP), which was influential for *adlan, stean, calan* and *adlvean,* and the other was on E05 (one SNP), with genes/QTL determining *stean, adlvean, pedan* and *fcol* (Figure 5).

**Table 2:** **Significant genotypic associations with anthocyanin content- and fruit color-related traits**

Trait	Marker	Chrom.	Position (cM)	Association group	p-value	q-value	PVE	MAF
*adlan*	10532_PstI_L317	E01	28.48	E01.1	5.04E-04	1.19E-02	8%	24.6%
	21901_PstI_L329	E02	58.27	E02.1	2.34E-03	4.80E-02	6%	19.9%
	24985_PstI_L311	E06	151.80	E06.1	3.76E-11	2.32E-09	22%	33.5%
	35442_PstI_L404	E10	69.13	E10.2	2.04E-05	5.24E-04	11%	48.7%
	15158_PstI_L379	E10	69.39	E10.2	4.08E-07	1.79E-05	13%	43.5%
	36033_PstI_L358	E11	68.04	E11.1	1.69E-05	4.74E-04	11%	42.4%
*stean*	27031_PstI_L365	E01	110.78	E01.2	3.25E-04	1.77E-02	7%	31.4%
	21901_PstI_L329	E02	58.27	E02.1	7.73E-04	2.85E-02	7%	19.9%
	12391_PstI_L355	E05	94.93	E05.1	1.05E-03	2.85E-02	7%	46.6%
	9226_PstI_L398	E08	1.80	E08.1	1.47E-03	3.63E-02	5%	19.4%
	35442_PstI_L404	E10	69.13	E10.2	5.51E-09	1.50E-06	18%	48.7%
	15158_PstI_L379	E10	69.39	E10.2	2.89E-05	3.92E-03	9%	43.5%
	19126_PstI_L349	E10	69.39	E10.2	2.08E-04	1.41E-02	7%	5.7%
	31471_PstI_L271	E10	70.39	E10.2	1.91E-03	3.99E-02	6%	30.4%
	3382_PstI_L285	E10	128.30	E10.3	9.56E-04	2.85E-02	6%	29.8%
	19601_PstI_L364	E10	128.34	E10.3	9.56E-04	2.85E-02	6%	29.8%
	33571_PstI_L387	E10	128.55	E10.3	9.56E-04	2.85E-02	6%	29.8%
*calan*	21901_PstI_L329	E02	58.27	E02.1	2.87E-05	2.71E-03	11%	19.9%
	31763_PstI_L370	E10	6.25	E10.1	3.37E-04	2.39E-02	8%	43.5%
	35442_PstI_L404	E10	69.13	E10.2	5.96E-12	1.69E-09	24%	48.7%
	15158_PstI_L379	E10	69.39	E10.2	5.12E-08	7.24E-06	15%	43.5%
*adlvean*	21901_PstI_L329	E02	58.27	E02.1	3.74E-04	1.48E-02	8%	19.9%
	25734_PstI_L387	E05	87.34	E05.1	1.51E-03	3.26E-02	7%	39.3%
	35442_PstI_L404	E10	69.13	E10.2	1.22E-09	2.91E-07	20%	48.7%
	15158_PstI_L379	E10	69.39	E10.2	2.41E-05	2.87E-03	9%	43.5%
	19126_PstI_L349	E10	69.39	E10.2	1.36E-03	3.23E-02	5%	5.7%
	3382_PstI_L285	E10	128.30	E10.3	1.68E-04	7.99E-03	7%	29.8%
	19601_PstI_L364	E10	128.34	E10.3	1.68E-04	7.99E-03	7%	29.8%
	33571_PstI_L387	E10	128.55	E10.3	1.68E-04	7.99E-03	7%	29.8%
	36033_PstI_L358	E11	68.04	E11.1	7.80E-04	2.10E-02	7%	42.4%
*ablvean*	35442_PstI_L404	E10	69.13	E10.2	8.16E-07	1.35E-04	14%	48.7%
	15158_PstI_L379	E10	69.39	E10.2	4.21E-07	1.35E-04	13%	43.5%
	31471_PstI_L271	E10	70.39	E10.2	7.03E-04	3.87E-02	7%	30.4%
	3382_PstI_L285	E10	128.30	E10.3	7.28E-06	4.81E-04	10%	29.8%
	19601_PstI_L364	E10	128.34	E10.3	7.28E-06	4.81E-04	10%	29.8%
	33571_PstI_L387	E10	128.55	E10.3	7.28E-06	4.81E-04	10%	29.8%
*pedan*	25734_PstI_L387	E05	87.34	E05.1	3.80E-06	4.28E-04	12%	39.3%
	35442_PstI_L404	E10	69.13	E10.2	2.05E-10	6.94E-08	20%	48.7%
	15158_PstI_L379	E10	69.39	E10.2	1.39E-09	2.35E-07	17%	43.5%
	3382_PstI_L285	E10	128.30	E10.3	1.90E-04	1.07E-02	7%	29.8%
	19601_PstI_L364	E10	128.34	E10.3	1.90E-04	1.07E-02	7%	29.8%
	33571_PstI_L387	E10	128.55	E10.3	1.90E-04	1.07E-02	7%	29.8%
*fcol*	27031_PstI_L365	E01	110.78	E01.2	4.66E-04	9.83E-03	7%	31.4%
	25776_PstI_L386	E05	100.27	E05.1	1.07E-04	4.53E-03	11%	20.4%
	34571_PstI_L286	E07	15.66	E07.1	2.60E-04	6.09E-03	6%	46.6%
	19381_PstI_L396	E10	64.21	E10.2	8.81E-05	4.53E-03	10%	49.2%
	35442_PstI_L404	E10	69.13	E10.2	1.22E-08	2.58E-06	22%	48.7%
	19126_PstI_L349	E10	69.39	E10.2	2.15E-04	5.67E-03	10%	5.7%
	3382_PstI_L285	E10	128.30	E10.3	1.51E-04	4.53E-03	6%	29.8%
	19601_PstI_L364	E10	128.34	E10.3	1.51E-04	4.53E-03	6%	29.8%
	33571_PstI_L387	E10	128.55	E10.3	1.51E-04	4.53E-03	6%	29.8%
	36033_PstI_L358	E11	68.04	E11.1	6.92E-06	7.30E-04	17%	42.4%
*fglo*	3687_PstI_L304	E03	104.00	E03.1	4.87E-04	3.88E-02	6%	23%
	3382_PstI_L285	E10	128.30	E10.3	1.07E-05	1.14E-03	10%	30%
	19601_PstI_L364	E10	128.34	E10.3	1.07E-05	1.14E-03	10%	30%
	33571_PstI_L387	E10	128.55	E10.3	1.07E-05	1.14E-03	10%	30%

The associated SNPs’ ID, genomic location, relevant association group, the significance of the association (both p- and q-values), PVE (phenotypic variability explained) and MAF (minimum allele frequency) are shown.

Detailed information regarding the individual genotype/phenotype associations is given in Table 2. The SNP locus most significantly associated with *adlan* was 24985_PstI_L311 (group 06.1), with a MAF of 33.5% and a PVE of 22%; for *stean, calan, adlvean, ablvean, pedan* and *fcol* 35442_PstI_L404 (group 10.2), with a MAF of 48.7% and a PVE of 14-24%. For *fglo*, three highly significantly associated SNPs were detected, namely 3382_PstI_L285, 19601_PstI_L364 and 33571_PstI_L387; these had a MAF of 30% and each had a PVE of 10%.

### Synteny with tomato and the identification of potential candidate genes

**Figure 6:**
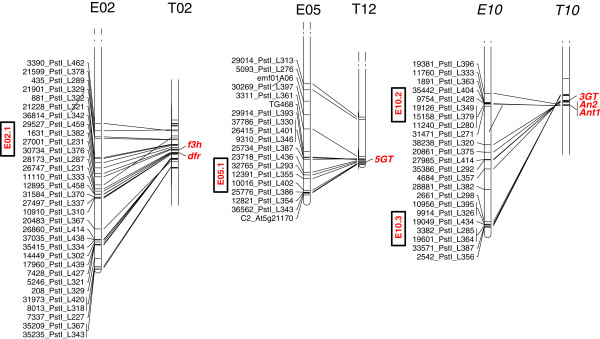
**Synteny between tomato and eggplant chromosomes.** The latter are shown on the left with bars indicating the site of QTL for anthocyanin content and fruit color. Their tomato orthologs are shown on the right, along with the position of possible candidate genes.

The regions of chromosomes E02, E05 and E10 harboring genetic factors underlying anthocyanin content were aligned with the tomato genome sequence. E02 and T02 are known to be syntenic, while part of E05 is syntenic with the lower section of T05 and the rest with the lower section of T12; E10 corresponds to the upper section of T05 and the lower one of T10 [42, 43]. Genes in tomato encoding flavonone 3-hydroxylase and dihydroflavonol 4-reductase are present on T02 in a region homologous with group E02.1, which harbors genes/QTL for *adlan, stean, calan* and *adlvean* (Figure 6). The location of the E05.1 group (*stean*, *adlvean*, *pedan* and *fcol*) corresponds to a segment of T12 in which a gene encoding the anthocyanin synthesis-associated enzyme UDP glucose anthocyanidin 5–0 glucosyltransferase is located (Figure 6). The tomato gene encoding UDP glucose anthocyanidin 3–0 glucosyltransferase and the two MYB transcription factors *Ant1* and *An2* are present on T10, in a region syntenic to groups E10.2 and E10.3; genes/QTL in these groups had a strong influence over the pigmentation of both the vegetative parts of the plant and the fruit.

## Discussion

### Genetic variation and population structure

The 191 members of the association panel were deliberately selected to represent the full range of phenotypic diversity in eggplant. The panel was genotyped with a subset of 384 of the >10,000 SNPs identified by sequencing RAD tags generated from the genomic DNA of 67/3 and 305E40, the parents of an established F_2_ mapping population [44]. Most of the SNPs included in the genotyping chip were unambiguously scorable and informative, producing average MAF and PIC values of 0.30 and 0.41, respectively. A small number of SNPs had to be discarded for reasons of poor scoring quality or low informativeness. Particularly low PIC values were obtained in the distal region of the chromosome E02 (Additional file 2: Figure S1B); this chromosomal region is the site of the locus *Rfo-sa1*, which confers resistance against the soil-borne fungus *Fusarium oxysporum* f. sp. *melongenae* which was introgressed in the parental line 305E40 from *Solanum aethiopicum* gr. *gilo* [45, 46]. Therefore, 305E40 carries some rather low frequency alleles which show a rather low frequency among the accession under study. The information provided by these polymorphisms are of interest only in the artificial progeny in which they are well represented while may result of scarce usefulness in a germplasm panel.

The population structure of the panel (Figure 1) comprised two rather distinct sub-populations, which broadly matched the accessions’ provenance. The species is thought to have been domesticated in Asia [47–49] and introduced into the Mediterranean basin by the Arabs in the 7^th^ to 8^th^ century CE [50]. The distinctness of the two gene-pools reflects a history of independent selection and adaptation to different environments and consumer preferences. When the PCO analysis was applied to just the “Occidental” entries no evidence of correlation between provenance and genetic relatedness was found, while a clustering related to the three morphological groups we previously described (i.e. group 1 -long, light and curved fruits-, group 2 -oblong shaped, medium weighted fruits- and group 3 -round an heavy fruits), was detected (Additional file 3: Figure S2B) [17]. Possibly, different uses of different types of fruits may have generated separate groups of varieties cultivated in different areas, with a different history of selection responsible of the observed genetic differentiation. The picture was rather different for the “Oriental” gene pool (Additional file 3: Figure S2A); the Asian material did form two recognizable clusters (Additional file 3: Figure S2A), with most of the Indian, SE Asian and Indonesian materials forming one group and the Chinese ones the other. This behavior replicated the outcome of a previous diversity study based on SSR markers [17], thereby further supporting the hypothesis that eggplant was domesticated independently in the Indian subcontinent and in China [47, 49].

### LD in eggplant

In order to account for population structure, two different corrections to the r^2^ measure were attempted, as proposed by Mangin et al. [25]. The estimate of LD derived without these corrections was unreliable, as it included apparent associations over long intra-chromosomal distances (Figure 2), and even between loci mapping to two different chromosomes (Figure 3). Applying the r^2^_s_ model reduced the extent of these clearly artefactual associations, but a more stringent method was still needed to correct for bias due to genetic relatedness. This was provided by the r^2^_sv_ model, which achieved a 30 fold reduction in associations between unlinked markers, leaving high LD values only between pairs of genetically linked SNP loci. The end estimate for the extension of LD was 3.4 cM, which matches reasonably well with the level reported for eggplant by Ge et al. [16], and also with those documented in other self-pollinating species such as the near-relative tomato (6–8 cM; [51]), *Arabidopsis thaliana* (10 kb; [52]), barley (3.5 cM; [26]) and wheat (1–5 cM; [27]). LD was not uniformly distributed along the genome (Figure 3), a phenomenon which has also been noted elsewhere [27, 51, 53, 54]. Its non-uniformity is thought to reflect the irregular distribution of recombination along the chromosome, but can also be influenced by positive selection [55]. Furthermore, the high level of LD and extended haplotype blocks in our material may be due to the high level of homozygosity which can hardly generate recombinations as well as the genetic bottleneck due to selection which drastically reduced the low frequent haplotypes and extended the association between markers. Such a level of LD is ideal for the GWA method, as it allows for an efficient coverage of the genome based on a relatively moderate number of markers, while still encouraging a high level of genetic diversity.

### GWA mapping of genetic factors underlying anthocyanin pigmentation and fruit color

QTL discovery in eggplant has to date been achieved using linkage mapping in bi-parental inter and intra-specific populations [6, 7, 10, 11, 56]. A first attempt to apply GWA has been published recently [16], in which 49 marker associations related to eight traits were reported. Some potentially fatal weaknesses in the analysis can, however, be identified. Firstly, the issue of a MAF threshold was not addressed, so it is not clear whether loci associated with a low MAF were discarded or included; the effect of their inclusion would be to generate false associations caused by the co-incidence of variation for a trait and a statistically under-represented allele. Secondly, the GLM model was used to estimate the significance of locus/trait associations, but this method has been shown to be incapable of adequately correcting for population structure [15, 57], unlike the MLM model used in the present study. Thirdly, no evidence of correcting for spurious associations (such as a q-q plot or a cumulative density function) was provided. Fourthly, a threshold false positive rate (p-value) of 0.01 was adopted as a threshold to validate the associations, instead of using a corrected threshold (e.g. the Bonferroni correction), or one for the false discovery rate.

The genetic basis of anthocyanin synthesis and accumulation has been widely explored in the *Solanaceae* [58–64]. In eggplant, this has long been thought to be rather complex [65, 66], involving at least three major and five minor loci, with the added complication of epistatic interactions and/or pleiotropic effects. The GWA procedure generated 56 associations between SNP loci and either anthocyanin content- or fruit color-related traits. According to Collard et al. [67], a QTL associated with a PVE of at least 10% should be considered as a “major” locus. There was a lack of any significant association involving *corcol* and *ablan*, but at least one “major” QTL was putatively identified for each of the other eight traits. The SNP loci associated with one (or more) traits were clustered into 12 groups, scattered over nine chromosomes. The extent of some of the inter-trait correlations suggests that what appeared to be a cluster of QTL is more likely a single pleiotropic locus, although the presence of a set of linked QTL cannot be definitively ruled out. Broad-sense heritabilities were generally >0.5 (the exceptions were *fglo* (0.38) and *corcol* (0.42)) with a limited genotype by environment effect, which confirms the proposition that the influence of the growing environment on anthocyanin pigmentation is quite limited [10, 68].

The genomic location of eight of the 12 association groups overlapped that of a known QTL, showing how effective GWA can be in identifying the genetic basis of quantitative traits. QTL identified via linkage analysis of bi-parental populations are generally considered to be experiment-specific, unless validated [69] and have often proven to be genetic background specific as well. Of the 20 SNP loci involved in the genotype/phenotype associations discovered here, six mapped to four genomic regions where no QTL related to anthocyanin content or fruit color has yet been reported (association groups E01.1, E03.1, E07.1 and E10.3; Figure 5, Table 3). Some of these markers could only have been identified thanks to the extent of the genetic variability which the GWA approach makes accessible; following a validation exercise, they may well prove to provide viable indirect selection tools in a practical breeding programme. The GWA study of Ge et al. [16] has located two marker/*fcol* associations, one on chromosome E01 and the other on E05, in correspondence to the association groups E01.2 and E05.1 carrying QTLs for the same trait.

**Table 3:** **Genotype/phenotype associations and known QTL location**

	GWA mapping		Family-based QTL mapping	
Chromosome	Association Group	Trait	QTL	Reference
E01	**E01.1**	***adlan***	-	-
	E01.2	*stean*	-	-
		*fcol*	fai1.1	Doganlar et al. [6]
			fap	Ge et al. [16]
E02	E02.1	*adlan*	lla2.2	Doganlar et al. [6]
		*stean*	steanE02.MT	Barchi et al. [10]
		*calan*	-	-
		*adlvean*	-	-
E03	**E03.1**	***fglo***	-	-
E05	E05.1	*stean*	steanE05.ML; steanE05.MT	Barchi et al. [10]
		*adlvean*	lveanE05.ML; lveanE05. MT	Barchi et al. [10]
		*pedan*	pedanE05.ML; pedanE05.MT	Barchi et al. [10]
		*fcol*	fap	Ge et al. [16]
E06	E06.1	*adlan*	ablanE06.ML	Barchi et al. [10]
			lla6.1	Doganlar et al. [6]
E07	**E07.1**	***fcol***	**-**	**-**
E08	E08.1	*stean*	-	-
E10	E10.1	*calan*	-	-
	E10.2	*adlan*	ablanE10a.ML; ablanE10a.MT	Barchi et al. [10]
			lla10.1	Doganlar et al. [6]
		*stean*	steanE10.ML; steanE10.ML	Barchi et al. [10]
			sa10.1	Doganlar et al. [6]
		*calan*	calanE10.ML; calanE10.MT	Barchi et al. [10]
		*adlvean*	lveanE10.ML; lveanE10.MT	Barchi et al. [10]
		*ablvean*	lveanE10.ML; lveanE10.MT	Barchi et al. [10]
		*pedan*	-	-
		*fcol*	fap10.1; fai10.1; fc10.1	Doganlar et al. [6]
	**E10.3**	***stean***	-	-
		***adlvean***	-	-
		***ablvean***	-	-
		***pedan***	-	-
		***fcol***	-	-
		***fglo***	-	-
E11	E11.1	*adlan*	ablanE11.ML	Barchi et al. [10]
E11		*adlvean*	-	-
E11		*fcol*	-	-

The four locations where no QTL related to anthocyanin content or fruit color have been located are shown in bold.

### Synteny and possible orthologs in other Solanaceae species

To date most of the effort invested in the genetic analysis of anthocyanin pigmentation in the *Solanaceae* has been focused on potato, sweet pepper and tomato. In the latter crop, 13 genes (some encoding enzymes and transcription factors) involved in anthocyanin synthesis have been described [62]. The detailed understanding of this pathway, along with the well-established syntenic relationships between the tomato and eggplant chromosomes [10, 42, 70], means that it is reasonable to search for candidate genes in eggplant by inspecting the gene content of the syntenic tomato sequence. Synteny was observed between E02.1 (harboring marker/trait associations for *stean, adlan,calan* and *adlvean*) and a portion of tomato chromosome T02, where two genes encoding enzymes (flavonone 3-hydroxilase and dihydroflavonol 4-reductase) involved in the anthocyanin production pathway reside. One of these has been identified as the gene underlying the *aw* (anthocyanin without) QTL, in the presence of which there is a complete absence of anthocyanin throughout plant development [71]. The same gene is responsible for the potato *R* QTL [72], which produces red pelargonidin-based anthocyanin pigments. E05.1 (harboring marker/trait associations for *adlvean, pedan, stean* and *fcol*) lies in a region syntenic to a portion of T12 which harbors *5GT,* a gene involved in the storage of betanidin (a fruit and flower pigment) in the vacuole [73]. E10.2 and E10.3 (harboring marker/trait associations for *ablvean, adlan, adlvean, calan, fcol, fglo, pedan* and *stean*) share synteny with a portion of T10 which carries several genes related to the anthocyanin production pathway, *3GT* and the two MYB transcription factors *ANT1* and *AN2* (a and b loci) [62]. *ANT1* regulates the genes encoding chalcone synthase and dihydroflavonol 4-reductase, key enzymes involved in the synthesis of anthocyanin compounds [74]. *ANT1* is considered to be the prime candidate for the *ag* (anthocyanin gainer) QTL responsible for the delayed expression of anthocyanin [70]. In potato, *AN2*a is the likely candidate for *QTL I,* responsible for tissue-specific anthocyanin expression [59, 75] and *AN2*b for *F*, a regulator of anthocyanin expression in the flower [58]. In sweet pepper, a MYB transcription factor encoded by *A* underlies the accumulation of anthocyanin pigment in the foliage, flower and immature fruit [61].

## Conclusion

The development of large-scale genotyping capacity has allowed the concept of GWA to become a viable approach for the genetic dissection of quantitative traits. Here, the technique has been applied to uncover the genomic regions harboring genes underlying anthocyanin pigmentation and fruit color traits in eggplant. The GWA mapping approach was effective in validating a number of established QTL and, thanks to the wide diversity captured by the panel of genotypes in study, was able to detect a series of novel marker/trait associations. Synteny with tomato has allowed the ready identification of candidate orthologues for the chromosome E02, E05 and E12 QTLs related to anthocyanin accumulation.

### Availability of supporting data

The data sets supporting the results of this article are available in the LabArchives repository at the following web address http://dx.doi.org/10.6070/H4NG4NK5.

## Electronic supplementary material

Additional file 1: Table S1: List of the accessions used for the association mapping study. (PDF 426 KB)

Additional file 2: Figure S1: **A)** SNP performance. Loci with a MAF <0.05 were excluded from the GWA analysis. **B)** SNP PIC values across chromosomes. The solid line represents the average genome-wide PIC, and the broken line the variation in PIC value across chromosome E02; note the particularly low informativeness of loci at the distal end of this chromosome. (PDF 233 KB)

Additional file 3: Figure S2: The genetic architecture of the components of the germplasm panel. **A)** PCoA of the EA accessions. Those of Chinese origin cluster to the right of the plot, separated from those of S and SE Asian origin. **B)** PCoA of the WA accessions cluster according to their fruit morphology: group 1 - long, light, curved fruits, group 2 – oblong fruits of intermediate weight, group 3 – round, heavy fruits as defined by Cericola et al. [17]. (PDF 266 KB)

Additional file 4: Figure S3: PVE values. *adlan* = adaxial leaf lamina anthocyanin; *stean* = Stem anthocyanin; *ablan* = abaxial leaf lamina anthocyanin; *calan* = calyx anthocyanin; *corcol* = corolla color; *adlvean* = adaxial leaf venation anthocyanin; *ablvean* = abaxial leaf venation anthocyanin; *pedan* = fruit peduncle anthocyanin; *fcol* = fruit color; *fglo* = fruit glossiness. Var(g) = genotypic variance; Var(m) = genotype by location variance; Var(n) = genotype by year variance; Var(e) = residual variance. (PDF 174 KB)

Additional file 5: Figure S4: Pearson’s inter-trait correlations (upper part of the matrix) and regression coefficients (lower part). The histograms included on the diagonal show the distribution of trait values (see also Table 1). (PDF 348 KB)
