# Supplementary material for: Linkage disequilibrium and genome-wide association analysis for anthocyanin pigmentation and fruit color in eggplant
Source: BMC Genomics. 2014 Oct 14;15(1):896. doi: 10.1186/1471-2164-15-896 (PMC4210512; doi:10.1186/1471-2164-15-896)
Supplement: Supplementary file 1 — Additional file 1: Table S1: List of the accessions used for the association mapping study. (PDF 426 KB) [file 12864_2014_6597_MOESM1_ESM.pdf]

Supplementary Table 1. List of the accessions used for the association mapping study.

ID	Accession Name	Origin	Areal	Morphological groups*
AM_001	Dadali	Indonesia	EA	1
AM_004	Cima viola	Italy	WE	1
AM_005	Bianca ovale	Italy	WE	2
AM_010	1F5 (9)	Breeding line	WE	2
AM_011	Bianca Sicilia	Italy	WE	2
AM_013	CCR3	Breeding line	WE	1
AM_014	Mel	Italy	WE	2
AM_015	Luga 063	Italy	WE	1
AM_016	Prosperosa	Italy	WE	3
AM_018	Lunga Violetta Cinese	China	EA	1
AM_021	Tal 1/1	Italy	WE	1
AM_022	Angiò 4	China	EA	1
AM_023	BLK 1269	Breeding line	WE	2
AM_024	GIC/ 27-9	Breeding line	WE	2
AM_025	Tina	Italy	WE	1
AM_026	DR2	Italy	WE	1
AM_028	TBE84 D	Breeding line	WE	2
AM_029	FanE13 D	Breeding line	WE	2
AM_030	FanE27 D	Breeding line	WE	2
AM_031	FanE63 D	Breeding line	WE	2
AM_032	SNL 534-11	India	EA	3
AM_033	SNL 533-8	India	EA	3
AM_034	SNL 600-1	India	EA	2
AM_035	Cin 01/ 24-6	China	EA	2
AM_036	Viola Cin-A-1	China	EA	2
AM_037	Violetta di toscana	Italy	WE	3
AM_038	Bellezza nera	Italy	WE	2
AM_040	Violetta di Metaponto	Italy	WE	3
AM_041	28-08/3 (23-09)	Breeding line	WE	3
AM_042	31-08/4 (25-09)	Breeding line	WE	3
AM_043	51-08/4 (29-09)	Breeding line	WE	3
AM_044	52-08/4 (30-09)	Breeding line	WE	3
AM_045	55-08/5 (31-09)	Breeding line	WE	3
AM_046	16-09 (*15-12)*	Breeding line	WE	3
AM_047	P621-08	Breeding line	WE	3
AM_048	P623-08	Breeding line	WE	3
AM_049	P645-08	Breeding line	WE	3
AM_050	P649-08	Breeding line	WE	3
AM_051	P612-08	Breeding line	WExEA	3
AM_052	P390	Breeding line	WExEA	3
AM_053	P328	Breeding line	WExEA	3
AM_054	P656-08	Breeding line	WE	3
AM_055	msp 73-08	Breeding line	WE	2
AM_056	S 1052-08	Breeding line	WE	1
AM_057	LI324/06	Italy	WE	1
AM_058	msp 36-08	Italy	WE	1
AM_059	msp 42-08	Italy	WE	1
AM_060	msp 30-08	Italy	WE	1
AM_062	msp 55-08	Italy	WE	1

AM_063	L422-08	Italy	WE	1
AM_064	L717-289	Italy	WE	1
AM_067	Uga	Italy	WE	2
AM_068	Tana	Italy	WE	1
AM_069	Bin 6	Italy	WE	2
AM_070	Floralba	Italy	WE	2
AM_071	Ind Min	India	EA	1
AM_072	SM5/2	Breeding line	WE	2
AM_073	SM5/13	Breeding line	WE	2
AM_074	SM5/22	Breeding line	WE	1
AM_076	S.Nicandro	Italy	WE	2
AM_086	LS 3805 minden	Japan	EA	2
AM_098	CIN6	China	EA	3
AM_099	CIN5	China	EA	3
AM_100	CIN7	China	EA	3
AM_102	CIN9	China	EA	3
AM_103	LS611	Japan	EA	2
AM_106	Naga-Ungu	Indonesia	EA	1
AM_114	N 258-4	India	EA	1
AM_121	Indom melanz	Indonesia	EA	2
AM_124	PI17	Italy	WE	2
AM_126	Almagro	Spain	WE	2
AM_127	Larga negra	Spain	WE	1
AM_128	Listada	Spain	WE	2
AM_129	Tolga	Algeria	WE	2
AM_133	Black Beauty	Italy	WE	2
AM_134	Viserba	Italy	WE	1
AM_135	Black Beauty	Italy	WE	2
AM_136	Tonda Violetta Firenze	Italy	WE	2
AM_137	Violetta Lunga Romagna	Italy	WE	1
AM_138	Barbentane	France	WE	1
AM_139	Lunga Marina	Italy	WE	1
AM_140	Tonda di Valence	France	WE	3
AM_141	Lunga Violetta Scura Cannellina	Italy	WE	1
AM_142	Tonda Black Beauty	Italy	WE	2
AM_143	Bellezza Nera	Italy	WE	2
AM_144	Lunga Violetta Napoli	Italy	WE	1
AM_146	Black Beauty	Italy	WE	2
AM_147	Violetta Mostruosa NY	Italy	WE	2
AM_148	Slim Jim	India	EA	1
AM_149	Tonda Violetta Scura Valence	France	WE	3
AM_150	Grossissima Violetta Firenze	Italy	WE	2
AM_151	Violetta Lunga	Italy	WE	1
AM_152	Tonda Bianca	Italy	WE	2
AM_153	Prosperosa	Italy	WE	3
AM_155	Daejang	China	EA	1
AM_156	Buia	Italy	WE	2
AM_157	Baffa	Italy	WE	2
AM_158	Ank2	India	EA	2
AM_159	CN2	China	EA	3
AM_160	Dourga	France	WE	2
AM_162	Tunisia Baharia	Italy	WE	3
AM_163	Pusa Purple Cluster	India	EA	1
AM_167	Angio 3	China	EA	1

AM_168	Angio 5	China	EA	2
AM_169	Bianca striata verde	Italy	WE	2
AM_170	SM19/14	Breeding line	WE	2
AM_171	Palermitana	Italy	WE	3
AM_173	Pusa Purple Long	India	EA	1
AM_174	JM (Slim Jim)	India	EA	1
AM_175	Cannellina Sarnense	Italy	WE	1
AM_176	Sita	Italy	WE	2
AM_177	FiL white	Turchia	WE	2
AM_178	Lunga napoli	Italy	WE	1
AM_179	1237/06	Italy	WE	1
AM_180	Listada Tacconi	Italy	WE	2
AM_181	Suraj(143)	India	EA	2
AM_182	Pusa Round	India	EA	2
AM_183	Chaojiuye Yuanquie	China	EA	3
AM_184	He Shanwang	China	EA	3
AM_185	TAI 440	Indochinese Region	EA	3
AM_187	Naveen	India	EA	2
AM_188	TAI 444	Indochinese Region	EA	2
AM_189	TAI 445	Indochinese Region	EA	1
AM_190	TAI 446	Indochinese Region	EA	1
AM_191	TAI 449	China	EA	3
AM_193	TAI 453	Indochinese Region	EA	2
AM_194	TAI 455	Thailand	EA	2
AM_195	TAI 456	Myanmar	EA	2
AM_196	TAI 457	India	EA	2
AM_198	TH 6413 Raos	Indonesia	EA	1
AM_199	TAI 470	Thailand	EA	2
AM_200	TAI 475	Thailand	EA	2
AM_201	TAI 477	Thailand	EA	2
AM_202	TAI 480	India	EA	2
AM_203	TAI 481	China	EA	1
AM_204	TAI 483	India	EA	1
AM_205	TAI 484	India	EA	1
AM_206	7 CN	China	EA	3
AM_207	9 CN	China	EA	3
AM_208	17 CN	China	EA	1
AM_210	67-3	Breeding line	WExEA	3
AM_211	305 E40	Breeding line	WE	1
AM_212	CGN17464 (PI 176759)	Turkey	WE	2
AM_213	CGN23345 (PI 169641)	Turkey	WE	2
AM_214	CGN18783 (Croisette)	France	WE	1
AM_215	CGN18531 (Patchem)	Turkey	WE	1
AM_217	CGN17449 (Topak; PI 175917)	Turkey	WE	2
AM_218	CGN17451 (Dolmalik; PI 176758)	Turkey	WE	2
AM_221	CGN17579 (PI 169648)	Turkey	WE	-
AM_222	CGN23346 (Topatan; PI 169649)	Turkey	WE	2
AM_224	CGN17581 (PI 169651)	Turkey	WE	1
AM_228	CGN23343 (PI 167328)	Turkey	WE	1
AM_230	CGN23344 (Bostan; PI 169639)	Turkey	WE	2
AM_231	CGN18591 (PI 171847)	Turkey	WE	2
AM_232	CGN18595 (PI 171852)	Turkey	WE	1
AM_233	CGN18779 (De Barbentane)	France	WE	1
AM_234	CGN23309 (Dolg; PI 358232)	Macedonia	WE	1

AM_235	CGN18484 (Morska Pata; PI 358242)	Macedonia	WE	1
AM_236	CGN18782 (Violette Longue Hative)	France	WE	1
AM_238	CGN17453 (Yesilkoy 27)	Turkey	WE	1
AM_240	CGN18578 (Kemer; PI 169655)	Turkey	WE	-
AM_241	CGN23348 (PI 169658)	Turkey	WE	1
AM_243	CGN18585 (PI 169663)	Turkey	WE	1
AM_249	CGN23351 (PI 174362)	Turkey	WE	2
AM_251	CGN24467 (Berenjena Listada)	Spain	WE	2
AM_252	CGN18505 (Berenjena Redonda)	Spain	WE	3
AM_253	CGN24468 (Caminal)	France	WE	1
AM_257	CGN18776 (Longue Hative)	France	WE	1
AM_258	CGN17456 (Monda)	France	WE	2
AM_259	CGN23315 (Ronde de Valence)	France	WE	3
AM_260	CGN17479 (Semiredonda Jaspeada)	Spain	WE	2
AM_262	CGN23772	Nigeria	WE	2
AM_264	Mezza Lunga Violetta	Italy	WE	1
AM_265	Lunghissima Precoce Violetta	Italy	WE	1
AM_266	Dingaras	China	EA	1
AM_268	L 129	Indonesia	EA	1
AM_269	Talindo	Indonesia	EA	1
AM_271	DS1	Breeding line	WE	2
AM_273	DS2	Breeding line	WE	2
AM_274	DS4	Breeding line	WE	2
AM_275	1 CAAS	China	EA	3
AM_278	4 CAAS	China	EA	3
AM_279	5 CAAS	China	EA	3
AM_284	10 CAAS	China	EA	1
AM_285	11 CAAS	China	EA	1
AM_288	14 CAAS	China	EA	2
AM_289	15 CAAS	China	EA	3
AM_290	16 CAAS	China	EA	3
AM_291	17 CAAS	China	EA	3
AM_292	18 CAAS	China	EA	1
AM_293	19 CAAS	China	EA	1

*Morphological groups as defined by Cericola et al. [17]
