# Supplementary figures and images for: Linkage disequilibrium and genome-wide association analysis for anthocyanin pigmentation and fruit color in eggplant
Source: BMC Genomics. 2014 Oct 14;15(1):896. doi: 10.1186/1471-2164-15-896 (PMC4210512; doi:10.1186/1471-2164-15-896)

Supplementary Figure 1

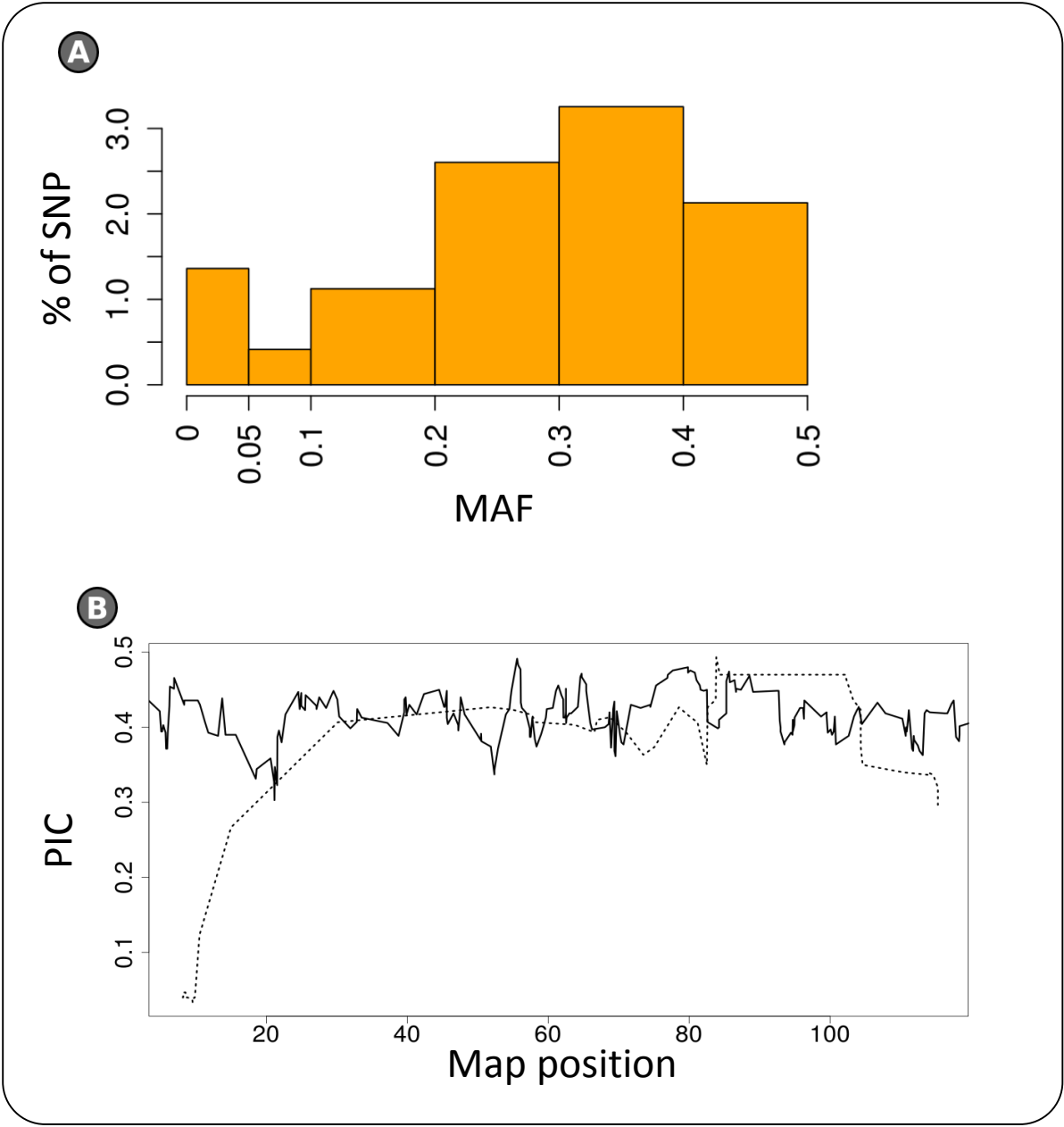

Supplement: Supplementary file 2 — Additional file 2: Figure S1: A) SNP performance. Loci with a MAF <0.05 were excluded from the GWA analysis. B) SNP PIC values across chromosomes. The solid line represents the average genome-wide PIC, and the broken line the variation in PIC value across chromosome E02; note the particularly low informativeness of loci at the distal end of this chromosome. (PDF 233 KB) [file 12864_2014_6597_MOESM2_ESM.pdf]

Supplementary Figure 2

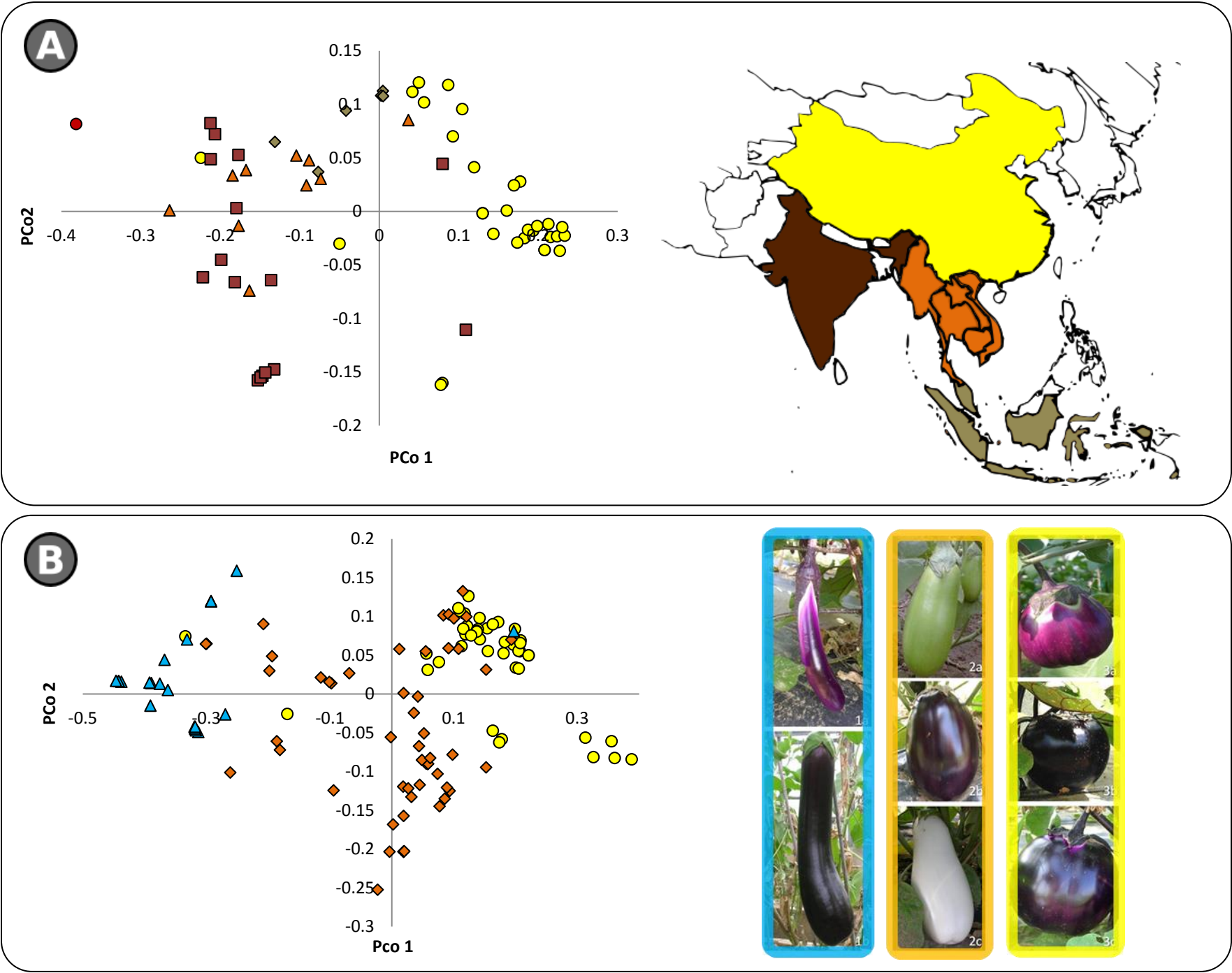

Supplement: Supplementary file 3 — Additional file 3: Figure S2: The genetic architecture of the components of the germplasm panel. A) PCoA of the EA accessions. Those of Chinese origin cluster to the right of the plot, separated from those of S and SE Asian origin. B) PCoA of the WA accessions cluster according to their fruit morphology: group 1 - long, light, curved fruits, group 2 – oblong fruits of intermediate weight, group 3 – round, heavy fruits as defined by Cericola et al. [17]. (PDF 266 KB) [file 12864_2014_6597_MOESM3_ESM.pdf]

Supplementary Figure 3

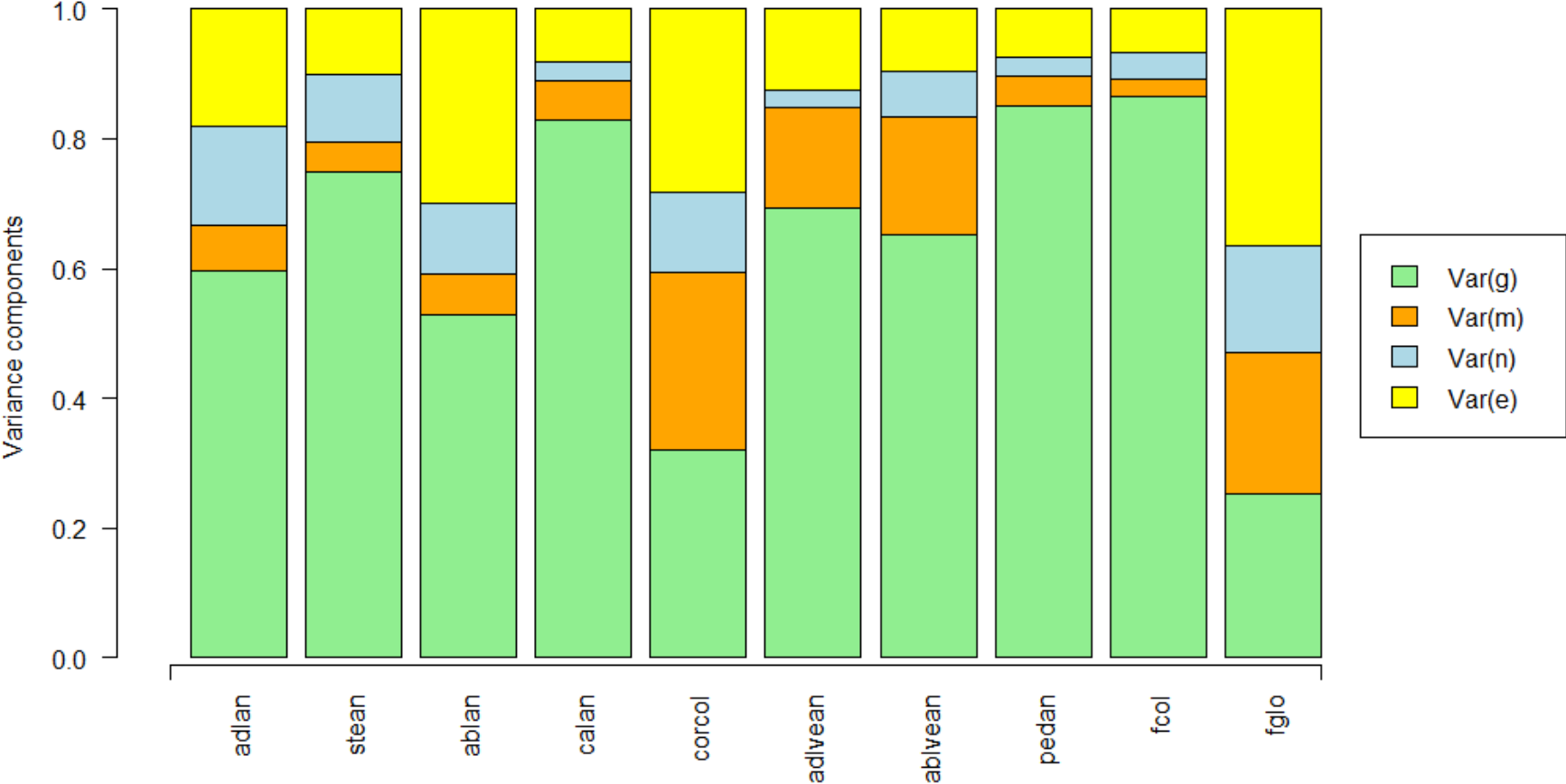

Supplement: Supplementary file 4 — Additional file 4: Figure S3: PVE values. adlan = adaxial leaf lamina anthocyanin; stean = Stem anthocyanin; ablan = abaxial leaf lamina anthocyanin; calan = calyx anthocyanin; corcol = corolla color; adlvean = adaxial leaf venation anthocyanin; ablvean = abaxial leaf venation anthocyanin; pedan = fruit peduncle anthocyanin; fcol = fruit color; fglo = fruit glossiness. Var(g) = genotypic variance; Var(m) = genotype by location variance; Var(n) = genotype by year variance; Var(e) = residual variance. (PDF 174 KB) [file 12864_2014_6597_MOESM4_ESM.pdf]

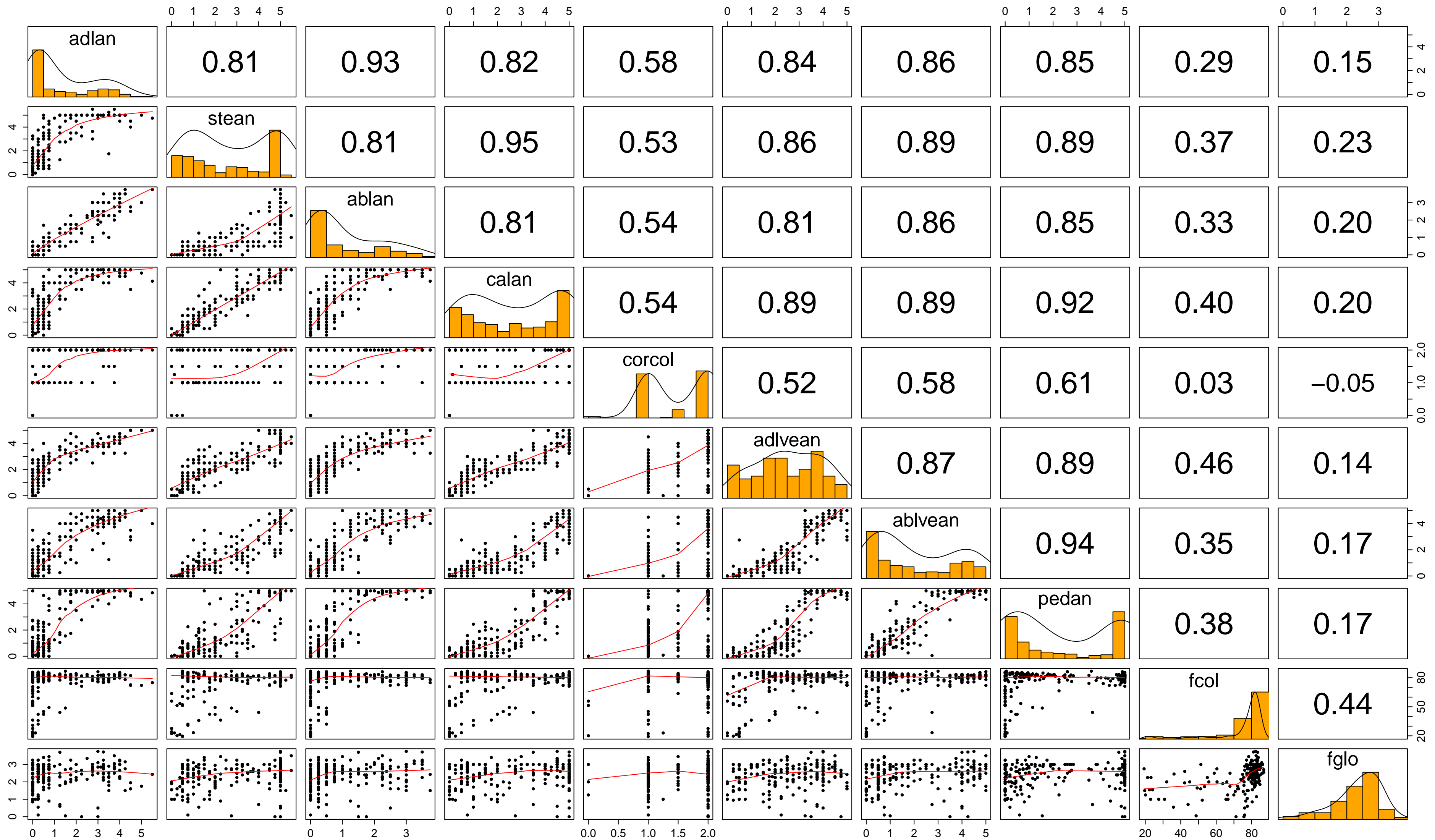

Supplement: Supplementary file 5 — Additional file 5: Figure S4: Pearson’s inter-trait correlations (upper part of the matrix) and regression coefficients (lower part). The histograms included on the diagonal show the distribution of trait values (see also Table 1). (PDF 348 KB) [file 12864_2014_6597_MOESM5_ESM.pdf]
